# Radiation effects on antitumor immune responses: current perspectives and challenges

**DOI:** 10.1177/1758834017742575

**Published:** 2018-01-18

**Authors:** Thomas Walle, Rafael Martinez Monge, Adelheid Cerwenka, Daniel Ajona, Ignacio Melero, Fernando Lecanda

**Affiliations:** Innate Immunity Group, German Cancer Research Center (DKFZ), Heidelberg, Germany; Department of Oncology Clínica Universidad de Navarra, Pamplona, Spain; German Cancer Research Center (DKFZ), Research Group Innate Immunity, Heidelberg, Germany; Division of Oncology, Centre for Applied Biomedical Research (CIMA), Pamplona, SpainIdiSNA, Navarra Institute for Health Research, Pamplona, SpainDepartment of Biochemistry and Genetics, University of Navarra, Pamplona, Spain Centro de Investigación Biomédica en Red de Cáncer (CIBERONC); Programme in Immunotherapy, Centre for Applied Biomedical Research (CIMA), Pamplona, SpainDepartment of Immunology and Immunotherapy, Clínica Universidad de Navarra, Pamplona, Spain Centro de Investigación Biomédica en Red de Cáncer (CIBERONC); Programme in Solid Tumours and Biomarkers, Division of Oncology, Centre for Applied Biomedical Research (CIMA), IdiSNA, Navarra Institute for Health Research, Department of Histology and Pathology, University of Navarra, School of Medicine, Pamplona, Spain. Centro de Investigación Biomédica en Red de Cáncer (CIBERONC)

**Keywords:** abscopal, brachytherapy, checkpoint inhibitors, immunogenic cell death, immunotherapy, PD-1, radiotherapy

## Abstract

Radiotherapy (RT) is currently used in more than 50% of cancer patients during the course of their disease in the curative, adjuvant or palliative setting. RT achieves good local control of tumor growth, conferring DNA damage and impacting tumor vasculature and the immune system. Formerly regarded as a merely immunosuppressive treatment, pre- and clinical observations indicate that the therapeutic effect of RT is partially immune mediated. In some instances, RT synergizes with immunotherapy (IT), through different mechanisms promoting an effective antitumor immune response. Cell death induced by RT is thought to be immunogenic and results in modulation of lymphocyte effector function in the tumor microenvironment promoting local control. Moreover, a systemic immune response can be elicited or modulated to exert effects outside the irradiation field (so called abscopal effects). In this review, we discuss the body of evidence related to RT and its immunogenic potential for the future design of novel combination therapies.

## Introduction

Radiotherapy (RT) represents one of the pillars in the management of cancer patients. Alone or in combination with surgery, RT displays a range of antitumor effects, which are mainly cytotoxic, evidenced by the drastic changes in proliferation, morphology and cell death, leading to tumor shrinkage. At the molecular level, RT induces nonrepairable DNA strand breaks, leading to mitotic catastrophe, resulting in cellular senescence and apoptosis.^1^ These cytotoxic effects can also affect leukocytes because conventional radiation fields frequently include the thymus, hematopoietic bone marrow or large blood volumes leading to lymphopenia together with impaired leukocyte function in irradiated cancer patients which perpetuates the view that RT is generally immunosuppressive.^2^

The first evidence for an immune-stimulatory effect of RT emerged from infrequent clinical observations of tumor remission outside the radiation field in satellite secondary tumors. This event was called the ‘abscopal effect’ (Latin, *ab scopus*, away from the target).^3^ Preclinical models showed that this effect is largely immune mediated,^3,4^ a finding further supported by associations in early clinical studies.^5–8^ Importantly, these abscopal effects seldom occur after RT alone, suggesting that RT as a single agent is not sufficient to trigger an effective antitumor immune response in cancer patients. Abscopal effects more frequently emerge in patients treated with combined RT and immunotherapy (IT).^8–11^ Likewise, RT boosted the antitumor effects of a range of immunotherapies including checkpoint inhibitors and adoptive transfers of T or natural killer (NK) cells. A wide range of ITs are currently being tested in combination with RT in clinical trials seeking to ameliorate current response and survival rates.^12^ Thus, the robust results obtained with IT and the RT-elicited abscopal effects open a new front to revolutionize the usage of RT.

In this review, we will delineate the scope of combined RT and IT, as well as recent advances in preclinical models and clinical trials showing the encouraging results of this dual combination. We will dissect the challenges of combining IT and RT, emphasizing the opportunities for increasing synergistic benefits.

## Significance and hurdles of radiation-induced immune responses

The combination of immune-checkpoint inhibitors with the ability of RT to act on the immune system has gathered much interest. Striking responses using checkpoint inhibition not previously anticipated in melanoma, lung and other solid tumors are leading to a paradigm shift, and represent novel US Food and Drug administration (FDA)-approved treatments for a growing number of tumor types.^13–15^ These drugs act by blocking negative regulators of T-cell activation, restoring antitumor activity that is usually impaired by tumor cells themselves and other elements present in the tumor microenvironment (TME). Unfortunately, checkpoint inhibitors are not always efficacious to induce tumor rejection, and a significant number of patients do not respond or become refractory to IT. Several obstacles preventing IT from unleashing its full potential have been proposed:

1. the insufficient priming of tumor-antigen reactive T cells;
2. the weak infiltration of antitumor effectors into the neoplastic tissues (lymphocyte exclusion phenotype);^16^
3. the presence of a highly immunosuppressive TME;
4. the ability of cancer cells to effectively evade recognition by immune effectors, impaired tumor-associated antigen presentation and the absence of danger-associated molecular patterns (DAMPs) and loss of sensitivity to interferon gamma (IFNγ).^17^

**Figure 1.**
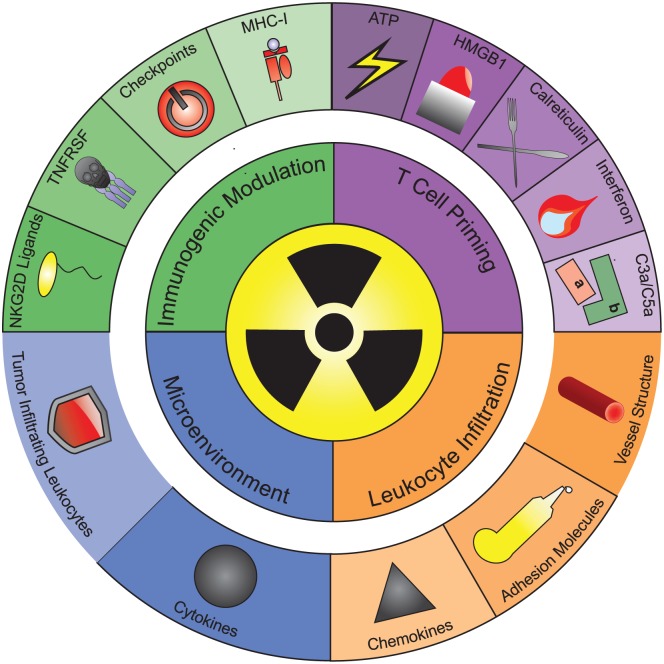
Principles of the radiation-induced immune response. The effects of RT on the immune system are conceptualized in four major organizing principles (inner circle): (a) the priming of TA-specific T cells; (b) leukocyte infiltration into the tumor tissue; (c) changes in the immunosuppressive TME; and (d) immunogenic modulation of the tumor cell phenotype, leading to increased sensitivity of irradiated tumor cells to lymphocyte-mediated lysis. The mechanisms involved in each of these organizing principles are displayed in the outer circle. (a) RT primes tumor antigen-specific T cells by inducing antigen uptake and maturation of dendritic cells. Five signals triggered by RT have been implicated in this process: the secretion of ATP and the alarmin HMGB1, the cell surface exposure of the eat-me signal calreticulin, radiation-induced interferons and activated complement fragments C5a/C3a. (b) RT drives leukocyte infiltration into the tumor tissue by three different mechanisms: changes in vessel structure, increased adhesion molecule expression on endothelium and the induction of chemokines. (c) RT also shapes the TME by triggering secretion of a plethora of cytokines and changing the presence and function of immunosuppressive leukocytes in the TME. (d) RT also modulates the immunophenotype of cancer cells by inducing the expression of MHC-I, ligands for the NKG2D receptor, ligands for immune checkpoint molecules and TNFRSF member Fas. These surface molecules increase or lower susceptibility of cancer cells to T and natural killer cell-mediated lysis. The different organizing principles are highly interconnected and influence each other’s occurrence and effect on tumor growth. ATP, adenosine triphosphate; HMGB1, high mobility group box; MHC-I, major histocompatibility complex I; NKG2D, natural killer cell lectin-like-receptor K1; RT, radiotherapy; TA, tumor antigen; TME, tumor microenvironment; TNFRSF, tumor necrosis factor superfamily.

The combination of RT with IT may offer novel strategies to overcome the current limitations. Using these limitations as organizing principles, we conceptualize the effects of RT on the antitumor immune response (Figure 1).

### Priming of tumor antigen-specific T cells

**Figure 2.**
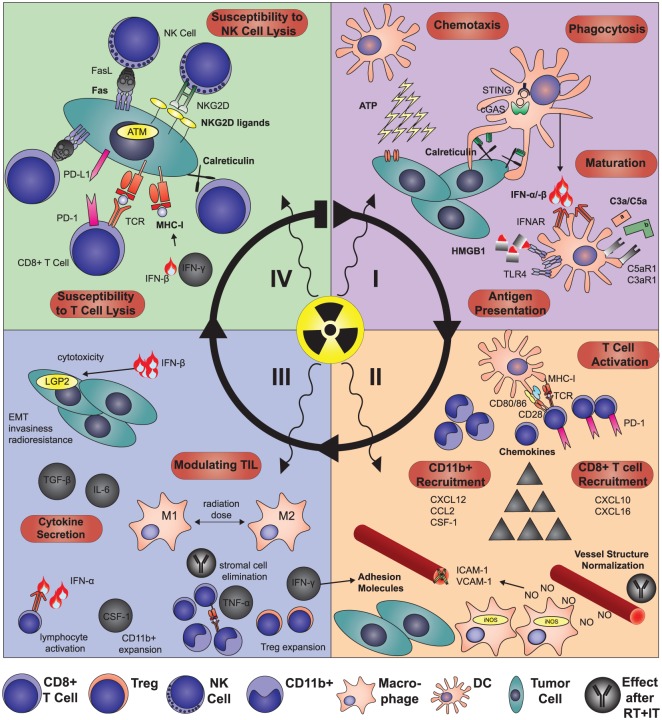
Mechanistic changes in the antitumor immune response after radiotherapy. (I) RT triggers the recruitment of DCs to the tumor site by inducing adenosine triphosphate release.^20–22^ Subsequently, calreticulin is translocated to the tumor cell surface, which triggers their phagocytosis.^23,24^ HMGB1 released after RT promotes processing and cross-presentation of tumor antigens taken up by DCs.^23,25^ Moreover, phagocytosis of irradiated tumor cells activates the cytosolic DNA sensing cGAS/STING pathway leading to the induction of IFN-β. This, together with complement activated by RT leads to DC maturation.^26–28^ (II) DCs then migrate to the tumor-draining lymph nodes and prime CD8+ T cells,^18,29^ which express high levels of PD-1, thus representing optimal targets for checkpoint inhibitors.^4,30,31^ In combination with IT, low-dose irradiation facilitates T-cell extravasation, which is mediated by iNOS+ macrophages and further perpetuated by the IFN-γ-dependent induction of adhesion molecules on the endothelium.^32,33^ After RT alone, immunosuppressive CD11b+ cells are recruited from the bone marrow and drive tumor regrowth and vasculogenesis and in an MMP-9-dependent manner.^34–36^ These CD11b+ myeloid cells are lured into the tumor tissue by radiation-induced CSF-1, CCL2 or CXCL12.^34,35,37–40^ Of note, the TME after RT fosters the secretion of CXCL12 by TGF-β and NO-mediated upregulation of HIF-1α.^38,41^ In contrast to these immunosuppressive chemokines, CXCL16 and CXCL9/10 can attract cytotoxic T cells and thereby enhance IT efficacy.^42–45^ (III) Once T cells activated by RT have infiltrated the tumor tissue, they encounter a heavily modified TME, which, in conjunction with IT, they can also modulate by killing immunosuppressive MDSCs by TNF-α or in a TCR-dependent manner.^46–48^ Radiation induces a plethora of cytokines including type I and II IFNs, which, next to their already-discussed functions, can directly activate leukocytes and have direct cytotoxic effects on tumor cells.^28,44,49^ However, several immunosuppressive cytokines are released into the TME post-RT such as TGF-β and IL-6 leading to epithelial–mesenchymal transition, invasiveness and radioresistance.^30,37,50^ IT helps to shift the post-RT cytokine milieu towards antitumor immunity. RT also alters IT efficacy by quantitative and qualitative changes in tumor-infiltrating immunosuppressive leukocytes. CD11b+ myeloid cells expand due to CSF-1 induction and depending on radiation-dose macrophages, are skewed towards an M1- or M2-like phenotype, with the latter being sequestered in hypoxic areas.^32,37,51–53^ In addition, Tregs accumulate due to priming by Langerhans cells and their intrinsic radioresistance.^54,55^ (IV) Finally, RT induces the expression of several molecules and receptors on the tumor cell surface, like MHC-I molecules,^56,57^ TNFR superfamily members,^57–60^ ATM-dependent induction of ligands for the NKG2D receptor^60–63^ and calreticulin,^23^ leading to enhanced tumor cell killing by CD8+ T and NK cells.^56,57,61,63^ However, RT can also induce excess levels of PD-L1 on tumor cells and thereby induce T-cell anergy underlining the rationale for combining RT and IT.^4,31,47,64–67^ ATM, ataxia teleangiectasia mutated; ATP, adenosine triphosphate; cGAS, cyclic GMP-AMP synthase; CCL, C-C motif chemokine ligand; CSF-1, colony stimulating factor-1; CXCL, C-X-C motif chemokine ligand; DC, dendritic cell; HIF-1α, hypoxia-inducible factor-1 alpha; HMGB1, high mobility group box 1; IFN, interferon; IL, interleukin; iNOS, nitric oxide synthase 2; IT, immunotherapy; LGP2, laboratories of genetics and physiology 2; M1, M1-like macrophage (iNOShi, Arg1lo, Fizz-1lo); M2, M2-like macrophage (iNOSlo Arg1hi, Fizz-1lo) MDSC, myeloid-derived suppressor cell; MHC-I, major histocompatibility complex I; MMP-9, matrix metalloproteinase 9; NK, natural killer cell; NKG2D, killer cell lectin-like receptor K1; NO, nitric oxide; PD-1, programmed-cell-death 1; PD-L1, programmed-cell-death ligand 1, CD274 molecule; RT, radiotherapy; STING, transmembrane protein 173; TCR, T cell receptor; TGF-β, transforming growth-factor beta, TME, tumor microenvironment; TNF, tumor necrosis factor; TNFR, tumor necrosis factor receptor; Treg, regulatory T cell.

Preclinical data show that RT-mediated tumor eradication largely depends on T cells and their ability to recognize tumor antigens with sufficient affinity.^18,19^ Irradiation, especially in combination with checkpoint inhibitors, effectively induces priming of tumor antigen-specific T cells in cancer patients and animal models. In the latter, T-cell priming mediates the rejection of established primary tumors and prevents distant dissemination. The mechanistic insights by which RT boosts tumor-specific immune responses are summarized in Figure 2.

A growing body of evidence indicates that RT-mediated T-cell priming occurs through the activation of different branches of host immunity. RT releases waves of potential tumor antigens in a phenomenon called ‘epitope spreading’ in which cell damage leads to the priming of tumor antigen-specific T cells, which attack the tumor, releasing another antigen wave creating a positive feedback loop.^68^ Interestingly, this process seems to be favored by checkpoint inhibitors, which enhance the repertoire of tumor antigen-specific T cells. In this context, RT could facilitate dendritic-cell-mediated tumor antigen-specific T-cell priming.^19,69,70^

In addition, RT triggers immunogenic cell death (ICD) in a range of animal models. This is a unique type of cell death characterized by the release of danger signals, which elicit the effective costimulation concomitant to presentation of tumor antigens and subsequent priming of antigen-specific T cells.^71^ Cellular events mediating effective ICD after RT include the release of ATP,^20^ which attracts dendritic cells (DCs) into the tumor,^21^ as well as the cell surface exposure of calreticulin, an endoplasmic reticulum-resident protein, which promotes phagocytosis of irradiated tumor cells.^72,73^ Finally, another factor is the release of the chromatin-binding protein HMGB1 (high mobility group box 1), which facilitates antigen presentation and type I-IFN-mediated DC maturation.^46^ Interestingly, low calreticulin and HMGB1 levels are associated with poor prognosis in RT-treated cancer patients. These findings substantiate the notion that RT could transform a tumor into an ‘*in situ* vaccine’ (Table 1).^74^ Importantly, a subset of DCs, now termed DC1 are critical for crosspriming of cytotoxic T lymphocytes including those involved in tumor immunity. These cells are specialized in taking up antigen from other cells and introducing the antigenic material into their class-I antigen-presenting pathway. Two studies have found that this rare basic leucine zipper ATF-like transcription factor 3 (BATF3) dependent DC subset is critical for the synergistic effects of RT and IT, including abscopal effects.^26,75^ In this line, it is proposed that DNA released from dying cells is able to turn on the transmembrane protein 173 (STING) pathway in tumor-surrounding DCs as a key element in the ignition of adaptive antitumor immunity.

**Table 1.** Mechanisms of radiation-induced T-cell priming.

Signal induced by RT	Function and mechanisms	Tumor	Dose	Time	References
**Calreticulin**	Is exposed on the surface of irradiated tumor cells Triggers phagocytosis of irradiated tumor cells by DCs Increases lysis of irradiated tumor cells by CTLs Is required for the formation of antitumor immunity after vaccination with irradiated tumor cells Correlates with OS in lung cancer patients receiving RT	3 × breast 1 × chordoma 2 × colon 2 × lung 1 × melanoma 2 × prostate	8 Gy 10 Gy 20 Gy 25 Gy 75 Gy 75 Gy	4h 7h 12h 24h 72h 4 d	Golden *et al*.;^21^ Garg *et al*.;^72^ Perez *et al*.;^73^ Gameiro *et al*.;^23^ Gameiro *et al*.;^76^ Obeid *et al*.^24^
**HMGB1**	Is released from irradiated tumor cells Triggers antigen presentation by DC and priming of antigen-specific T cells after local RT in a TLR4 dependent manner ESCC patients show elevated HMGB1 concentrations post CRT HMGB1 expression correlates with overall survival in ESCC patients receiving CRT	3 × breast 1 × ESCC 2 × lung 2 × lymphoma 1 × prostate	10 Gy 20 Gy 30 Gy 30–33*2 Gy 100 Gy	24h 48h 72h 72h 72h	Golden *et al*.;^21^ Gameiro *et al*.;^23^ Apetoh *et al*.;^25^ Suzuki *et al*.;^77^ Yoshimoto **et al*.*^78^
**ATP**	Is released from irradiated tumor cells Release from irradiated tumor cells is dependent on expression of the autophagy factor ATG5	1 × breast 1 × colon 1 × lung 1 × prostate	4 Gy 20 Gy 100 Gy	24h 24h 72h	Ko *et al*.;^22^ Golden *et al*.;^21^ Gameiro *et al*.^23^
**IFN-α/β**	Is induced by sensing of irradiated tumor cells in DCs in a cGAS and STING dependent manner Is required for priming of antigen-specific T cells by DCs after local RT and for the antitumor effect of RT Directly activates lymphocytes after RT Increases intratumoral IFN-γ production and CXCR3-dependent T-cell recruitment after local RT	1 × colon 1 × lung 2 × melanoma	14 Gy 15 Gy 20 Gy 20 Gy	48h 48h 72h NA	Wu *et al*.;^46^ Deng *et al*.;^26^ Lim *et al*.;^44^ Burnette *et al*.^79^
**Complement**	Is activated by local RT Complement activation can either favor or inhibit antitumor immune responses depending on the use of single dose or fractionated RT, respectively Triggers DC maturation and IFN- γ production in tumor-infiltrating CD8+ T cells after local RT	1 × breast 1 × colon 1 × lymphoma 1 × melanoma	20 Gy 4*1.5 Gy	24h 24h	Surace *et al*.;^27^ Elvington *et al*.^80^

Preclinical studies analyzing the mechanisms of antigen-specific T-cell priming after RT, as well as studies analyzing the effect of RT on DC maturation and antigen-presentation. Indicated are the analyzed tumor types and the lowest radiation dose and earliest timepoints after RT at which maximum effects on the indicated mechanism were observed *in vivo* or *in vitro*.

ATG5, autophagy related 5; ATP, adenosine triphosphate; cGAS, cyclic GMP-AMP synthase; CTL, cytotoxic lymphocytes; CRT, chemoradiotherapy; CXCR, C-X-C motif chemokine receptor; d, days; DC, dendritic cell; ESCC, esophageal squamous cell carcinoma; Gy, Gray; HMGB1, high mobility group box 1; h, hours; IFN, interferon; NA, not applicable; n.d., not disclosed; OS, overall survival; RT, radiotherapy; STING, transmembrane protein 173; TLR4, toll-like receptor 4.

Current approved ITs directed to restore antitumor immune responses might benefit from the antitumor effect of RT because of debulking and because of enhancing crosspriming of T cells. For instance, the relevance of RT in priming antitumor-specific T-cell responses is also supported by the high expression of programmed-cell-death ligand 1 (PD-L1) in irradiated tumors.^47^ RT enhances the expression of CD137, a co-stimulatory immune checkpoint molecule on tumor and programmed-cell-death 1 (PD-1) on T cells.^4^ Consequently, PD-1 or PD-L1 blockade and CD137 activation act in synergism with RT and favor abscopal effects.^81^ These preclinical findings suggest that local RT may enhance the systemic beneficial effects of immunostimulatory monoclonal antibodies and explain the large number of ongoing clinical trials exploring the clinical activity of these combinations^82^ (*vide infra*).

### Attracting leukocytes into the tumor tissue

Low infiltration of effector T cells into the tumor represents a major obstacle for cancer IT.^83,84^ RT-mediated leukocyte infiltration can be directed by changes in leukocyte extravasation, an event partially modulated by the *in situ* generation of leukocyte chemoattractants. In addition to effector T cells, RT also induces the infiltration of a wide range of leukocytes including NK cells, regulatory T cells (Tregs) and CD11b-positive (CD11b+) cells, such as MDSCs (myeloid-derived suppressor cells) and TAMs (tumor-associated macrophages).

RT by itself exerts dual and opposite effects on the immune system, which underscores its role as a double-edged sword in the antitumor immune response. On the one hand, RT increases tumor infiltration by endogenously primed or adoptively transferred effector T cells, NK cells and other leukocytes which impede tumor growth.^32,85^ On the other hand, RT increases infiltration by Treg and CD11b+ cells, including MDSCs and TAMs, which are associated with an immunosuppressive TME and poor outcome in cancer patients.^46,86^ However, CD11b+-mediated immunosuppression may be transient and be later replaced by influx of effector T cells.^87^ Moreover, in combined RT with IT, the accumulation of CD11b+ cells can be prevented and the immunostimulatory effects of RT seem to prevail.^48^ For example, intratumor vaccination and monoclonal antibodies against PD-L1 can render CD11b+ cells susceptible to T-cell mediated lysis.^46^ In the same line, MDSCs and Tregs can be directly depleted using monoclonal antibodies, targeting CD11b or CD25 to achieve more salient effects.^54,85^ These findings highlight the notion that radiation-induced immune responses can be optimized using novel combined strategies to achieve an optimal therapeutic synergy.

An important mechanism involved in leukocyte infiltration after RT is the alteration and normalization of the aberrant tumor vasculature. Tumors induce a chronically activated angiogenesis creating anomalous vasculature, resulting in distorted vessel sprouting, abnormal branching, large vessel diameter, abnormal blood flow with leakiness, and microhemorrhaging. In addition, an endothelium nonpermissive for lymphocytes is maintained by an array of immunosuppressive and proangiogenic signals together with endothelium-associated cells.

The combination of RT and IT leads to a normalization of the vasculature characterized by a reduction of vascular density and leakiness, together with increased vessel homogeneity. This phenotypic change is associated with higher infiltration by endogenous or transferred CD8+ T cells and higher immunotherapeutic efficacy. Some of these effects are mediated by nitric oxide (NO) that, depending on radiation dose, can exert dual functions. At least after low-dose (LD) radiation, normalization of vasculature can be mediated by the induction of nitric oxide synthase (iNOS) by macrophages residing in the irradiated tissue, an event crucial for the therapeutic efficacy of adoptive T-cell transfer.^32^ However, when high-dose RT is used without concurrent IT, the tumor-promoting role of NO prevails over its effect on vasculature.^41^

In addition to changes in tumor vasculature, RT also induces the expression of adhesion molecules on blood vessel and lymphatic endothelial cells, which are crucial mediators for migration and extravasation of leukocytes into the tumor bulk.^75,88^ So far, their functional relevance in modifying antitumor immunity post-RT remains to be established. Radiation-induced intercellular adhesion molecule 1 (ICAM-1), for instance, mediates the transmigration of tumor-promoting CD11b+ myeloid cells after RT alone.^85^ Nevertheless, when RT was combined with an adoptive T-cell transfer or a cancer vaccine, induction of adhesion molecules was associated with higher infiltration by cytotoxic T cells and therapeutic efficacy.^89^ Intriguingly, RT-induced vascular cell adhesion molecule 1 (VCAM-1) expression depends on nitric oxide synthase 2 (iNOS) positive macrophages and on interferon-γ (IFN-γ) produced by hematopoietic cells.^32,33^ It is therefore likely that radiation-induced mechanisms of T-cell priming and T-cell infiltration are closely interconnected.

Among the most relevant signals regulating leukocyte infiltration post-RT are radiation-induced chemokines secreted by irradiated tumor cells and other stromal components, including myeloid cells and fibroblasts. The net balance and the type of radiation-induced chemokines determine the composition of the leukocyte infiltrate. For instance, RT-induced chemokine (C-X-C motif) ligand 9 (CXCL9), -10 and -16 secretion attracts adoptively transferred T cells and thereby enhances tumor control.^42–45^ By contrast, CXCL12 and colony stimulating factor-1 (CSF-1) induced by RT can attract tumor-promoting CD11b+ myeloid cells.^34,35^ Concurrently, this massive release of chemokines can also potently increase epithelial–mesenchymal transition and invasiveness of tumor cells.^90,91^ Thus, the overall combination of RT-induced chemokines will determine not only the infiltration of pro- or antitumorogenic leukocytes, but will also affect tumor cell behavior.

Beyond these effects on vessel structure and chemokine expression, RT can also lead to the accumulation of Tregs in the tumor tissue postradiation due to their high intrinsic radioresistance^54^ and due to Treg priming by radioresistant Langerhans-cells.^55^

In summary, RT can help endogenous CD8+ T cells or transferred CD8+ T cells and NK cells to infiltrate the tumor tissue and thereby enhance IT efficacy. Radiation-induced changes in the tumor vasculature generally support tumor regrowth after RT alone by favoring infiltration of immunosuppressive myeloid cells. Importantly, IT counteracts this radiation-induced accumulation of immunosuppressive leukocytes in the tumor and thereby prevents tumor regrowth after RT by increasing vascular permeability to cytotoxic lymphocytes. Further comprehensive studies are needed to dissect how the chemokine milieu can be optimally influenced by RT to support IT efficacy (Table 2).

**Table 2.** Mechanisms of radiation-induced leukocyte infiltration.

Effect of RT	Function and mechanisms		Tumor	Dose	Time	References
	Favoring tumor growth	Favoring tumor control				
Reduction of vascular density and induction of vasculogenesis	CD11b+ cells are recruited by RT and drive vasculogenesis and tumor regrowth in an MMP-9-dependent manner	Vessel normalization is mediated by iNOS+ M1-like macrophages and leads to enhanced infiltration by endogenous or adoptively transferred CD8+ and CD4+ T cells RT in combination with IT normalizes the vasculature: vessel area and hemorrhages decrease, circularity and pericyte coverage increase Decreased vessel density after RT is associated with impaired tumor growth in irradiated tissues	1 × breast 1 × colon 1 × glioma 2 × HNSCC 2 × lung 2 × pancreas (endocrine) 1 × sarcoma	2 Gy 7.5 Gy 9.5 Gy 10 Gy 15 Gy 20 Gy 20 Gy 15 Gy + 6 Gy	24 h 3 d 8 d 14 d 14 d 17 d n.d. n.d.	Klug *et al*.;^32^ Ahn *et al*.;^85^ Mondini *et al*.;^89^ Kozin *et al*.;^34^ Kioi *et al*.;^35^ Ganss *et al*.;^92^ Udagawa *et al*.;^93^ Ahn *et al*.^36^
Induction of adhesion molecules on tumor vasculature	ICAM-1 is induced on irradiated endothelial cells and mediates leukocyte adhesion to irradiated endothelium; Radiation-induced ICAM-1 expression is associated with transmigration of tumor-promoting CD11b+ myeloid cells after RT alone and with higher infiltration by tumor antigen-specific T cells after RT + IT; ICAM-1 is induced by RT on tumor-associated endothelium of HNSCC patients	VCAM-1 induction after RT + IT is mediated by iNOS+ M1-like macrophages and IFN-γ from hematopoietic cells; VCAM-1 induction by RT is associated with higher infiltration of CD8+ T cells	2 × HNSCC 2 × melanoma 1 × pancreas (endocrine) NA NA	2 Gy 7.5 Gy 10 Gy 15 Gy 15 Gy 20 Gy 30*2 Gy	24 h 24 h 24 h 6 d 7 d 7 d n.d.	Klug *et al*.;^32^ Ahn *et al*.;^85^ Mondini *et al*.;^89^ Lugade *et al*.;^33^ Handschel *et al*.;^94^ Lugade *et al*.;^95^ Hallahan *et al*.^96^
Induction of chemokines	CXCL12: Radiation-induced CXCL12 recruits CD11b+ myeloid cells that mediate vasculogenesis; Radiation-induced fibroblast-derived CXCL12 increases tumor-cell EMT and invasiveness; CXCL12 induction is mediated by upregulation of HIF-1α Other: Radiation induces CSF-1 in tumor cells, which attracts MDSCs and macrophages favoring tumor growth; RT induces CCL2 and CCL5 expression in an IL-6-dependent manner, which attracts monocytes and favors tumor growth; CCL2-mediated attraction of monocytes favors tumor growth after RT	CXCL9, 10: STING-dependent induction of type I IFNs mediates upregulation of CXCL10 and subsequent T cell, macrophage and DC infiltration; CXCL9, CXCL10 and CCL4 correlate with response to RT + anti-CTLA-4 in melanoma patients Other: Radiation-induced CXCL16 attracts T and NK cells and increases antitumor activity of adoptively transferred T cells; Radiation-induced CCL5 is associated with increased CD8+ T-cell infiltration	3 × breast 1 × colon 2 × glioma 1 × HNSCC 2 × lung 2 × melanoma 3 × pancreas 1 × prostate	4 Gy 6 Gy 8 Gy 15 Gy 15 Gy 15 Gy 20 Gy 20 Gy 20 Gy 20 Gy 20 Gy 2*12 Gy 18–50 Gy	24 h 24 h 24 h 48 h 48 h 48 h 72 h 72 h 7 d 8 d 14 d n.d. n.d.	Hiniker *et al*.;^5^ Deng *et al*.;^26^ Matsumura *et al*.;^42^ Yoon *et al*.;^43^ Lim *et al*.;^44^ Zheng *et al*.;^45^ Kozin *et al*.;^34^ Kioi *et al*.;^35^ Li *et al*.;^90^ Xu *et al*.;^37^ Tabatabai *et al*.;^38^ Kalbasi *et al*.;^39^ Wang *et al*.^40^

Representative preclinical/clinical studies analyzing the effects of RT on radiation-induced leukocyte infiltration. Indicated are effects of RT leading to leukocyte infiltration, suggested underlying mechanisms, the analyzed tumor type, the lowest radiation dose and earliest timepoint after RT at which maximum effects were observed *in vivo* or *in vitro* (if no *in vivo* data available).

CCL, C-C motif chemokine ligand; CSF, colony stimulating factor; CTLA-4; cytotoxic T-lymphocyte associated protein 4; CXCL, C-X-C motif chemokine ligand; d, days; EMT, ephithelial-mesenchymal transition; HIF, hypoxia inducible factor; HNSCC, head and neck squamous cell carcinoma; Gy, Gray; h, hours; ICAM-1, intercellular adhesion molecule 1; IL, interleukin; IFN, interferon; IT, immunotherapy; iNOS+, nitric oxide synthase 2; MDSCs, myeloid-derived suppressor cells; MMP-9, matrix metalloproteinase; M1, M1-like macrophage (iNOShi, Arg1lo, Fizz-1lo); M2, M2-like macrophage (iNOSlo, Arg1hi, Fizz-1hi); NA, not applicable; n.d., not disclosed; NK, natural killer; RT, radiotherapy; STING, transmembrane protein 173; VCAM-1, vascular cell adhesion molecule 1.

### Modifying the tumor microenvironment

#### Modulation by secreted factors of the tumor microenvironment

Once tumor-reactive lymphocytes have been primed and have infiltrated the tumor tissue, they must overcome a highly immunosuppressive tumor milieu. The TME encompasses an intricate interplay of tumor cells and their associated stroma, which, as the tumor progresses, entails the secretion of an array of soluble factors (Table 3). RT profoundly alters the TME, impacting tumor growth and effective antitumor immune responses. A wide variety of growth factors and cytokines is released after RT into the tumor milieu to configure a net balance of pro- and anti-immunogenic cues, greatly modulating the immune response.

**Table 3.** Radiation-induced changes in the tumor microenvironment.

Effect of RT	Function and mechanisms		Tumor	Dose	Time	References
	Favoring tumor growth	Favoring tumor control				
Cytokine secretion	TGF-β: RT induces TGF-β facilitating metastasis formation; TGF-β abolishes priming of antigen-specific T cells in response to RT; TGF-β confers intrinsic protection from radiation damage to cancer cells; Captopril inhibits induction of TGF-β by RT in endothelial cells IL-6: IL-6 is released from tumor cells after RT and increases tumor cell invasiveness CSF-1: CSF-1 drives systemic expansion of CD11b+ cells	Type I IFN: Radiation-induced IFN-β leads to upregulation of MHC-I expression on tumor cells; IFN-α/β induced by RT directly activates lymphocytes by the IFNAR; Tumor cells protect themselves from IFN-β mediated killing by upregulation of LGP2 Type II IFN: IFN-γ is induced by irradiation and mediates the antitumor effect of RT; Radiation-induced IFN-γ from hematopoietic cells mediates radiation-induced upregulation of VCAM-1 on tumor vasculature and radiation-induced MHC-I expression on tumor cells	4 × breast 1 × colon 1 × glioma 1 × HNSCC 1 × lung 2 × melanoma 1 × prostate NA	8 Gy 10 Gy 10 Gy 12 Gy 15 Gy 15 Gy 15 Gy 14*2 Gy 5*6 Gy 3*12 Gy	24 h 24 h 4 d 5 d 7 d 7 d NA NA NA n.d.	Lugade *et al*.;^33^ Lim *et al*.;^44^ Coppe *et al*.;^91^ Vanpouille-Box *et al*.;^30^ Vanpouille-Box *et al*.;^97^ Xu *et al*.;^37^ Biswas *et al*.;^98^ Wang *et al*.;^99^ Wei *et al*.;^100^ Widau *et al*.^49^
Macrophage polarization	M2: High dose RT skews macrophages towards an M2 phenotype, in an NFkB dependent manner, due to cell intrinsic effects or due to soluble factors released from irradiated tumor cells M2-like macrophages from high dose irradiated tumors promote tumor growth; High-dose RT triggers iNOS induction in macrophages favoring tumor growth; Macrophage polarization after RT depends on macrophage genotype	M1: Low dose RT skews macrophages towards an iNOS+ producing M1-like phenotype by cell intrinsic mechanisms; iNOS+ M1-like macrophages from low dose irradiated mice potentiate the therapeutic effect of adoptive T cell transfers; iNOS+ M1-like macrophages from low dose irradiated mice in combination with adoptively transferred T cells normalize the tumor vasculature	2 × breast 1 × glioma 2 × pancreas (endocrine) 3 × prostate NA	2 Gy 3 Gy 2*2 Gy 4 Gy 6 Gy 10 Gy 25 Gy 25 Gy 25 Gy	24 h 24 h 24 h 72 h 5 d 14 d 21 d 3 W 4 W	Klug *et al*.;^32^ Li *et al*.;^41^ Xu *et al*.;^37^ Tsai *et al*.;^51^ Prakash *et al*.;^101^ Crittenden *et al*.;^52^ Chiang *et al*.;^102^ Chen *et al*.;^53^ Coates *et al*.^103^
Immunosuppressive leukocytes	Priming of Treg cells after RT by radioresistant Langerhans cells facilitates tumor growth; RT leads to increased abundance of tumor infiltrating Tregs, due to their intrinsic radioresistance	RT triggers antigen-presentation on MDSCs leading to their eradication by CD8+ T cells or TNF-α	1 × breast 1 × lung 1 × melanoma 1 × prostate 1 × sarcoma	6 Gy 10 Gy 10 Gy 12 Gy 14 Gy	48 h 48 h 72 h 10 d 12 d	Wu *et al*.;^46^ Deng *et al*.;^47^ Kachikwu *et al*.;^54^ Price *et al*.^55^

Representative preclinical/clinical studies analyzing the effects of RT on the tumor microenvironment. Indicated are effects of RT on the tumor microenvironment, suggested underlying mechanisms, the analyzed tumor type and the lowest radiation dose and earliest time-point after RT at which maximum effects were observed *in vivo* or *in vitro* (if no *in vivo* data available).

CSF, colony stimulating factor; d, days; Gy, Gray; h, hours; HNSCC, head and neck squamous cell carcinoma; IFN, interferon; IFNAR, interferon-α receptor; IL, interleukin; iNOS+, nitric oxide synthase 2; IT, immunotherapy; LGP2, laboratories of genetics and physiology 2; MDSC, myeloid-derived suppressor cell; MHC, major histocompatibility complex; M1, M1-like macrophage (iNOShi, Arg1lo, Fizz-1lo); M2, M2-like macrophage (iNOSlo, Arg1hi, Fizz-1hi); NA, not applicable; n.d., not disclosed; NFκB, nuclear factor kappa B; PD-L1, CD274 molecule; TGF, transforming growth factor; TNF, tumor necrosis factor; RT, radiotherapy; Treg, regulatory T lymphocytes; VCAM-1, vascular cell adhesion molecule 1; W, weeks.

RT induces a cytokine burst from a few hours post-RT to several weeks postradiation. Cytokines are produced by both tumor cells and other tumor-associated cells including fibroblasts, macrophages and other leukocytes. The bulk of soluble mediators and cytokines that are released from senescent cells after cytotoxic treatments has been termed the senescence-associated secretory phenotype (SASP) and includes major secretion of interleukin-1β (IL-1β), IL-6, IL-7 and granulocyte-macrophage colony-stimulating factor (GM-CSF).^91^

Among the RT-mediated cytokine burst, transforming growth-factor beta (TGF-β) represents a major immunosuppressive factor limiting both the priming of tumor-reactive T cells and the release of macrophage pro-inflammatory cytokines. Indeed, this cytokine released after RT displays a protumorigenic and prometastatic role in some tumors.^30^ TGF-β release occurs in advanced pro-inflammatory and postradiation fibrotic events during tissue repair and extracellular matrix remodeling.^104^ Beyond its immunosuppressive effects, TGF-β also confers intrinsic radioresistance on tumor cells thus providing a dual protection from both the RT-induced cytotoxic effects, as well as antitumor immune response post-RT.^97^ In addition to transcriptional induction of TGF-β1, the activation of the latent as a result of exposure to ionizing radiation has been reported.^30,105^

CSF-1 is another RT-induced cytokine with a protumorigenic effect, which can drive the systemic expansion and survival of macrophages and MDSCs.^37^ In addition, IL-6 released from tumor cells’ T cells and TAMs supports tumor proliferation, invasiveness and radioresistance.^50^

Radiation-induced IFNs are crucial for the therapeutic effect of RT.^79^ They can directly activate T cells and other lymphocytes.^33,44^ The induction of type-I IFNs by RT *via* STING induces the priming of T cells, which in turn release IFN-γ.^26^ This cytokine seems to represent the dominant effector molecule of the antitumor immune response post-RT.^33^ Indeed, IFN-γ knockout mice showed severely diminished survival post-RT accompanied with low CD8+ T cell and high MDSC infiltration.^87^

Nevertheless, exposure to type I and II IFN signaling can also confer resistance to anti-CTLA-4 checkpoint blockade by upregulating PD-L1 or ligands for T-cell-inhibitory receptors suppressing antitumor immunity.^106^ Preventing chronic upregulation of these IFN-stimulated genes represents a highly effective approach to restore susceptibility of tumors that recur after combined RT and checkpoint blockade therapy.

RT can also activate the complement system, an event most likely mediated by the binding of immunoglobulin M (IgM) to necrotic cells.^27^ However, its impact on tumor control remains elusive. Whereas one-time activation of complement by single-dose RT led to improved tumor control and the induction of an adaptive antitumor immune response,^27^ repeated activation by fractionated RT showed a negative effect on tumor control.^80^

In contrast to local tumor irradiation, total body irradiation (TBI) leads to systemic changes in cytokine levels. This has been attributed to the removal of so-called ‘cytokine sinks’, which are host leukocytes sequestering and limiting the availability of cytokines.^107,108^ Thereby, TBI can drive the proliferation and engraftment of transferred CD8+ T cells and NK cells.^85,109^ Effects on intestinal permeability and ensuing translocation of luminal bacteria to the submucosa are also likely elements in the boosting of immunity by sublethal TBI.^110^

#### Modulation by changes in tumor infiltrating leukocytes

MDSCs and TAMs are considered protumor stromal components.^94^ RT can induce cross-presentation of tumor antigens on CD11b+ myeloid cells leading to their elimination by antigen-specific T cells.^48^ In addition, LD irradiation can skew macrophages towards an M1-polarized phenotype (iNOShi, Arg1lo, Fizz-1lo), including the upregulation of iNOS and T-helper-1 cytokines and render them supportive of antitumor immunity.^32^ However, higher radiation doses may polarize macrophages to an M2-phenotype that can promote tumor growth, an event mediated by soluble factors released from irradiated tumor cells.^51^ Since polarization of macrophages is extremely dependent on the contextual signals of the TME, characterization of the radiation-induced factors regulating polarization remains to be elucidated.

### Increasing tumor cell susceptibility to lymphocyte-mediated cytotoxicity

RT increases the susceptibility of tumor cells to T and NK-cell-mediated lysis by modulating the expression pattern of surface molecules including (major histocompatibility complex I) MHC-I, NK cell ligands, costimulatory receptors and death receptors. All these changes mediated by RT in immunomodulatory surface molecules, also observed with other cytotoxic treatments, have been termed immunogenic modulation.^111,112^

Radiation-induced upregulation of MHC-I molecules was associated with enhanced lysis of irradiated tumor cells by tumor antigen-specific T cells *in vitro* and *in vivo* (Table 4). The induction of MHC-I after RT occurs by a three-step mechanism, including a proteasome-dependent increase in cytosolic peptide levels, mTOR-dependent protein translation and induction of radiation-specific peptides.^56^ In addition to these cell intrinsic mechanisms of MHC-I induction, radiation-induced IFN-γ induces MHC-I upregulation.^33^ Of note, upregulation of MHC-I post-RT does not seem to be a universal mechanism, but it is confined to a fraction of tumor cell lines.^57^ Thus, RT could increase MHC-I levels in some tumors with low endogenous MHC-I to increase immune-mediated attack.

**Table 4.** Effects of ionizing radiation on tumor-cell susceptibility to T or natural killer cell-mediated lysis.

Signal induced by RT	Function and mechanisms	Tumor	Dose	Time	References
MHC-I/Ib	RT induces MHC-I expression on tumor cells in an IFN-β-dependent or mTOR-dependent manner; MHC-I induction on tumor cells is associated with increased susceptibility to T-cell-mediated lysis; MHC-I induction is associated with increased intracellular peptide levels; HLA-E induction on endothelial cells is associated with decreased susceptibility to NK-cell-mediated lysis	2 × colon 2 × lung 1 × melanoma 1 × prostate NA	4 Gy 10 Gy 3*12 Gy 25 Gy	12 h 18 h 72 h 5 d	Reits *et al*.;^56^ Garnett *et al*.;^57^ Wang *et al*.;^99^ Riederer *et al*.^113^
NKG2D ligands	Upregulation of NKG2D ligands after irradiation is mediated by ATM and the absence of STAT3 and can be inhibited by allopurinol; RAE-1 induction by RT restores intratumoral lymphocyte arrest and increases tumor control after combined anti-CTLA-4 and RT; MICA/B and ULBP1 induction is associated with increased NK cell mediated lysis; MICA/B is preferentially upregulated on stem-like cancer cells;	1 × breast 1 × glioma 1 × ovarian 2 × pancreas 2 × sarcoma	8 Gy 2*12 Gy 25 Gy 40 Gy 40 Gy	16h 16h 24h 24h 48h	Gasser *et al*.;^61^ Xu *et al*.;^62^ Ames *et al*.;^60^ Bedel *et al*.;^114^ Ruocco *et al*.^63^
TNFRSF members	RT induces Fas expression on stem-like cancer cells; Fas induction increases tumor cell susceptibility to T-cell mediated lysis and is associated with increased NK-cell-mediated lysis; Upregulation of CD137 is associated with enhanced tumor control by anti-CD137	3 × colon 1 × breast 1 × AML 2 × lung 1 × prostate 2 × sarcoma	8 Gy 8 Gy 10 Gy 16 Gy 20 Gy 20 Gy 20 Gy	24h 24h 48h 48h 72h 72h 7d	Garnett *et al*.;^57^ Chakraborty *et al*.;^77^ Chakraborty *et al*.;^59^ Ames *et al*.^60^; Shi and Siemann^115^
Immune checkpoint molecules	PD-L1 is upregulated on leukocytes and nonleukocytic cells in the tumor microenvironment and associated with enhanced tumor control after RT + anti-PD-L1; Radiation-induced PD-L1 expression limits T-cell proliferation and cytotoxicity; PD-L1 upregulation on tumor cells is mediated by CD8+ T cells and IL-6; High IL-6 expression correlates with PD-L1 expression in ESCC patients undergoing chemoradiotherapy; PD-1 is upregulated on T cells/TILs after RT	1 × bladder 2 × breast 2 × colon 1 × ESCC 1 × pancreas	6 Gy 9 Gy 5*2 Gy 12 Gy 12 Gy 15 Gy 20 Gy 30 Gy	24h 24h 48h 48h 72h 72h 72h 21d	Rodriguez-Ruiz *et al*.;^4^ Deng *et al*.;^47^ Verbrugge *et al*.;^31^ Azad *et al*.;^64^ Dovedi *et al*.;^65^ Liang *et al*.;^116^ Chen *et al*.;^66^ Wu *et al*.^67^
Other	Upregulation of the NKp30 ligand B7-H6 sensitizes tumor cells to NK-cell-mediated lysis; Radiation-induced calreticulin sensitizes tumor cells to CTL-mediated lysis	1 × AML 1 × breast 1 × lung 1 × prostate	8 Gy 10 Gy	24h 72h	Gameiro *et al*.;^23^ Cao *et al*.^117^

Representative preclinical/clinical studies analyzing the effects of RT on tumor-cell susceptibility to T or NK-cell-mediated lysis. Indicated are the respective effects of RT, the suggested underlying mechanisms, the analyzed tumor type and the lowest radiation dose and earliest timepoint after RT at which maximum effects on the indicated mechanism were observed *in vivo* or *in vitro* (if no *in vivo* data available).

CD137, tumor necrosis factor receptor superfamily member 9; AML, acute myeloid leukemia; ATM, ataxia telangiectasia mutated; NKp30 ligand B7-H6, natural killer cell cytotoxicity receptor 3 ligand 1; CTL, cytotoxic lymphocyte; CTLA-4, cytotoxic T-lymphocyte associated protein 4; d, days; ESCC; esophageal squamous cell carcinoma; Fas, Fas cell surface death receptor; Gy, Gray; HLA, human leukocyte antigen; IFN, interferon; IL, interleukin; MHC, major histocompatibility complex; MICA/B, MHC class I polypeptide-related sequence A OR MHC class I polypeptide-related sequence B; mTOR, mechanistic target of rapamycin; NA, not applicable; n.d., not disclosed; NK, natural killer; NKG2D, killer cell lectin-like receptor K1; NKp30, natural cytotoxicity triggering receptor 3; PD-1, programmed cell death 1; PD-L1, CD274 molecule; RAE-1, retinoic acid early inducible-1; RT, radiotherapy; STAT3, signal transducer and activator of transcription 3; TIL, tumor-infiltrating leukocyte; TNFRSF, tumor necrosis factor receptor superfamily; ULBP1, UL16 binding protein 1; W, weeks.

A crucial role of NK cell-mediated response eliminating small tumors and metastases has been shown. Irradiated tumors increase their visibility to NK cell-mediated cytotoxicity by enhanced expression of tumor ligands for NK receptors (NKG2D and NKp30) (Table 4).^61,118^ Although RT has shown beneficial effects on NK effector function, various factors in the TME can suppress NK effector responses. These include TGF-β, suppressive cells (MDSCs and Tregs), low pH and oxygen levels. Moreover, MHC-I molecules inhibit NK cell effector function even though they are crucial in the initiation of T-cell responses, as previously mentioned.

Other radiation-induced changes include the induction of Fas and TNF related apoptosis-inducing ligand receptors (TRAILRs) on tumor cells, members of the TNFR superfamily, which increases susceptibility to NK and T-cell-mediated lysis. Calreticulin is exposed in the outer layer of the plasma membrane upon irradiation and triggers tumor cell phagocytosis by DCs and increases susceptibility to T-cell-mediated lysis.^23,58,59,119^ Moreover, RT also induces expression of immune checkpoint ligands such as PD-L1 on tumor cells, which interferes with the effector functions of interacting T cells.^4,31,47^

## Implications for clinical radioimmunotherapy

### Clinical trials combining radio- and immunotherapy

Despite the plethora of preclinical information available, clinical data on combining RT and IT are scarce and largely limited to anti-CTLA-4 (ipilimumab)–RT combinations (Table 5). Importantly, concurrent or sequential combinations of RT and anti-CTLA-4 or anti-PD-1 were safe and well tolerated in several prospective clinical trials and retrospective analyses,^5,69,120–122^ although 34% grade 3 toxicities were reported when biologically effective doses of over 90 Gy for liver or lung stereotactic body radiation therapy (SBRT) were used.^6^

**Table 5.** Treatment characteristics of studies combining radiotherapy and checkpoint inhibitors.

Study type	Therapy	Tumor type	Timepoint of IT before/after RT	RT type	Fractionation (# fractions)	Cumulative dose (Gy)	Ref
**Phase III**	Anti-CTLA-4	Prostate	+<2 d	SBRT	1	8	Kwon *et al*.^123^
**Phase I/II**	Anti-CTLA-4	Prostate	+3 d	SBRT	1	8	Slovin *et al*.^121^
**Phase I**	Anti-CTLA-4	Melanoma	Concurrent	SBRT/IMRT/3D	1–5/10–15/ 5–15	18–50/30–45/20–40	Hiniker *et al*.^5^
**Phase I**	Anti-CTLA-4	Melanoma	+3–5 d	SBRT	2–3	12–24	Twyman-Saint Victor *et al*.^69^
**Phase I**	Anti-CTLA-4	Various	Concurrent	SBRT	4–10	50–60	Tang *et al*.^6^
**Retrospective**	Anti-CTLA-4	Melanoma	Concurrent/before/after	SRS (+ WBRT)	1–6 (+10–15)	16–24 (+30–37.5)	Skrepnik *et al*.^124^
**Retrospective**	Anti-CTLA-4	Melanoma	Concurrent/before/after	SRS	1	20	Mathew *et al*.^125^
**Retrospective**	Anti-CTLA-4	Melanoma	Concurrent/before/after	SRS	1	15–24	Kiess *et al*.^126^
**Retrospective**	Anti-CTLA-4	Melanoma	Concurrent/before/after	IMRS	3–5	15–21	Patel *et al*.^127^
**Retrospective**	Anti-CTLA-4	Melanoma	Concurrent/before/after	SRS	1–5	n.d.	Tazi *et al*.^128^
**Retrospective**	Anti-CTLA-4	Melanoma	Concurrent	CEBRT	n.d.	n.d.	Koller *et al*.^129^
**Retrospective**	Anti-CTLA-4	Melanoma	Concurrent	SBRT	1–25	24–62.5	Barker *et al*.^130^
**Retrospective**	Anti-CTLA-4	Melanoma	Before/after	SRS/WBRT	1–5/10–13	14–24/30–37.5	Silk *et al*.^131^
**Retrospective**	Anti-CTLA-4	Melanoma	Before/after	SBRT/CEBRT	1–5/6–16	18–25/21–42	Qin *et al*.^132^
**Retrospective**	Anti-CTLA-4 or anti-PD-1	Melanoma	Concurrent/before/after	SRS	1	15–24	Ahmed *et al*.^133^
**Retrospective**	Anti-CTLA-4 or anti-PD-1 or both	Melanoma, NSCLC, Renal cell	Concurrent/before/after	SBRT/SRS/IMRT/WBRT	1–15	8–66	Bang *et al*.^122^
**Retrospective**	Anti-PD-1	Melanoma	Concurrent/before: 1–6M/after: 1–6M	SRS	1–5	16–30	Ahmed *et al*.^134^
**Retrospective**	Anti-PD-L1	Various	Concurrent	SRS/3D	1–10	6–92 (BED)	Levy *et al*.^135^
**Preclinical**	Anti-CTLA-4	Breast	0 d/concurrent	Local	1–3	8–30	Vanpouille-Box *et al*.^136^
**Preclinical**	Anti-CTLA-4	Breast	+1 d	Local	1–2	12–24	Demaria *et al*.^81^
**Preclinical**	Anti-CTLA-4	Breast	+1 d	Local	2	24	Pilones *et al*.^137^
**Preclinical**	Anti-CTLA-4	Breast	+1 d	Local	2	24	Matsumura *et al*.^42^
**Preclinical**	Anti-CTLA-4	Breast	+2 d	Local	2	24	Ruocco *et al*.^63^
**Preclinical**	Anti-CTLA-4	Breast, colon	Concurrent	Local	1–5	20–30	Dewan *et al*.^138^
**Preclinical**	Anti-CTLA-4	Lung	+1 d	Local	1	30	Yoshimoto *et al*.^78^
**Preclinical**	Anti-CTLA-4	Lung, colon	−7 d	Local	1	20	McGinnis *et al*.^139^
**Preclinical**	Anti-CTLA-4 Anti-CD137	Glioma	Concurrent/1–2 d	Local	1	10	Belcaid *et al*.^140^
**Preclinical**	Anti-PD-L1	Breast	+21 d	Local	1	15	Liang *et al*.^116^
**Preclinical**	Anti-PD-L1	Breast, colon	+0 d	Local	1	12	Deng *et al*.^47^
**Preclinical**	Anti-PD-L1	Pancreatic	0 d/concurrent	Local	1–5	12–15	Azad *et al*.^64^
**Preclinical**	Anti-PD-L1 + cancer vaccine	Pancreatic	Concurrent	Local	2	35	Zheng *et al*.^45^
**Preclinical**	Anti-PD-L1 Anti-CD137	Breast, colon, melanoma	Concurrent	Local	2–3	16–24	Rodriguez-Ruiz *et al*.^141^
**Preclinical**	Anti-PD-L1 Anti-PD-1	Melanoma, colon	Concurrent	Local	5	10/20	Dovedi *et al*.^65^
**Preclinical**	Anti-PD-1	Breast	+1 d	Local	5	30	Vanpouille-Box *et al*.^142^
**Preclinical**	Anti-PD-1	Glioma	+0 d	Local	1	10	Zeng *et al*.^143^
**Preclinical**	Anti-PD-1	Lung	Concurrent	Local	3	36	Wang *et al*.^99^
**Preclinical**	Anti-PD-1	Melanoma	+5 d	Local	2	24	Hettich *et al*.^144^
**Preclinical**	Anti-PD-1	Renal cell	Concurrent	Local	1	15	Park *et al*.^145^
**Preclinical**	Anti-PD-1 Anti-CD137 Anti-CD40	Breast	Sequential/concurrent	Local	1–4	12–20	Verbrugge *et al*.^31^
**Preclinical**	Anti-PD-1 Anti-TIM-3	Glioma	Concurrent	Local	1	10	Kim *et al*.^146^
**Preclinical**	CD137 aptamer, anti-CTLA-4, anti-PD-1	Breast, colon sarcoma	+3–6 d	Local	1	12–20	Schrand *et al*.^147^
**Preclinical**	Anti-CD137	Breast, lung	+0 d/concurrent	Local	1–5	5–20	Shi and Siemann^115^
**Preclinical**	Anti-CD40	Lymphoma	+0 d	TBI	1	5	Honeychurch *et al*.^148^
**Preclinical**	Anti-CD134	NSCLC	+1 d	Local	3	60	Gough *et al*.^149^

Representative clinical and preclinical *in vivo* studies combining RT and checkpoint inhibitors. We define concurrent administration as ⩾1 dose of checkpoint inhibitor before and ⩾1 dose after a fraction of RT.

BED, biologically effective dose; CEBRT, conventional external beam radiation therapy; CTLA-4; cytotoxic T-lymphocyte associated protein 4; d, days; IMRS, intensity-modulated radiosurgery; IMRT, intensity-modulated radiation therapy; IT, immunotherapy; local, local radiotherapy (all preclinical radiation therapy techniques confined to a specified target volume of the animal); M, months, n.d., not disclosed; NSCLC, non-small cell lung cancer; PD-1, programmed cell death 1; PD-L1, CD274 molecule; RT, radiotherapy; SBRT, stereotactic body radiation therapy SRS, stereotactic radiosurgery; TIM-3, hepatitis A virus cellular receptor 2; TBI, total body irradiation; WBRT, whole-brain radiotherapy; 3D, three-dimensional conformal-radiation therapy.

Combinations of RT and ipilimumab have yielded encouraging results, especially in melanoma. A phase I clinical trial of concurrent ipilimumab and a physician’s choice RT regimen in 22 metastatic melanoma patients reported three complete responses (CRs) (13.6%).^5^ Notably, CRs are rare under ipilimumab monotherapy (1.4–2.2%).^13,150,151^ Another phase I trial in 22 melanoma patients of ipilimumab administered after RT did not show CRs but four partial responses (PRs) (18%), which is comparable with ipilimumab monotherapy (9.5–16.8%).^13,151,152^ Moreover, combined ipilimumab and SBRT achieved a 10% response rate in 35 patients with tumor types other than cutaneous melanoma.^6^ Nevertheless, in metastatic prostate cancer, a phase III clinical trial comparing ipilimumab (*n* = 399) and SBRT for bone metastases with SBRT alone (*n* = 400) failed to meet its primary endpoint.^123^ Notably, prostate cancer infrequently responds to immune checkpoint therapy and a synergy of RT and IT in preclinical models of metastatic prostate cancer has not been demonstrated yet; calling for more basic immunological research in this tumor type (Table 5).^17,153^ Aside from checkpoint inhibitors, a phase I/II trial combining GM-CSF and conformal RT in various tumor types reported an overall response rate of 26%, including two CRs in non-small cell lung cancer (NSCLC).^8^

Currently, more than 90 clinical trials assessing RT–IT combinations are ongoing.^82^ Of interest are combinations of RT with PD-1 antibodies, which in monotherapy have already shown clinical activity in a variety of cancers.^14,15,154,155^ Over 40 clinical trials are assessing safety and efficacy of this combination, including two phase III studies in glioblastoma multiforme and NSCLC [ClinicalTrials.gov identifiers: NCT02768558 and NCT02617589].^82^ Moreover, triple combinations of RT, anti-CTLA-4 and anti-PD-L1 are being tested and may have complementary effects on antitumor immune responses, as demonstrated in preclinical models [ClinicalTrials.gov identifiers: NCT02701400 and NCT02639026].^69^

Despite encouraging results in first clinical trials, most patients do not respond to RT–IT combinations. Several factors of the radiation regimen could be important to enhance its local and systemic antitumor effects in combination with IT.

### Dose of radiotherapy

Radiation dose largely affects both the immunomodulatory and cytotoxic effects of RT. Most preclinical studies combining RT and IT use high cumulative radiation doses of 5–20 Gy and most immune-stimulatory effects of RT peak at similar doses (Tables 1–5). For example, cytokine therapy combined with 10 Gy of local RT led to a strong synergistic effect and tumor control in 70% of mice, while combinations with 5 Gy or 2 Gy only led to tumor control in 50% or 10% of mice, respectively.^156^ Clinical trials combining RT and IT applied even higher cumulative doses up to 66 Gy using more hyperfractionated radiation regimens (Table 5). Interestingly, a preclinical study showed that radiation doses above 12–18 Gy attenuate the immunogenicity by cytosolic DNA degradation induced by exonuclease Trex1, whereas lower doses rather stimulate IFN-β secretion, activating a subset of DCs critically important for CD8 T-cell priming, allowing tumor rejection (abscopal effect) when combined with immune-checkpoint blockade.^136^ LD irradiation has been shown to have immunomodulatory capacity both when applied locally or as TBI. In 30 patients with low-grade B-cell lymphoma or mycosis fungoides, local LD irradiation of 2*2 Gy in combination with local administration of a toll-like receptor 9 (TLR9) agonist led to one CR and eight PRs at distant sites.^7,157^ In preclinical models, a local LD irradiation with 2 Gy synergized with adoptive T-cell transfer *via* the induction of iNOS in TAMs^32,158^ and also resulted in an abscopal effect when combined with an FMS-like tyrosine kinase 3 ligand.^3^ Moreover, 1.25 Gy total body LD irradiation in combination with a DC gp100 tumor vaccine enhanced priming of antigen-specific T cells and reduced relative Treg numbers in peripheral lymph nodes.^158,159^ Furthermore, total body LD irradiation with 0.1, 0.2 Gy or 0.75 Gy has been repeatedly shown to reduce outgrowth of intravenously injected tumor cells in the lungs of different mouse models, an event associated with increased NK cell numbers and cytotoxicity.^160–164^ However, the dose range in which the beneficial effects of total body LD irradiation can be observed appears to be narrow and slightly higher doses can already abrogate NK cell proliferation and activity.^163^ The advantage of local LD radiation results from its mild adverse events facilitating clinical application.^165^ Several ongoing clinical trials are investigating the immunomodulatory properties of local LD irradiation in pancreatic, colorectal and NSCLC patients.^166–168^

### Fractionation of RT

Fractionation of RT represents another key factor usually applied to reduce radiation damage to healthy tissues and maximize exposure of tumor cells in a sensitive phase of their cell cycle. Focused modern radiation techniques allow for a reduced number of RT fractions and prevent generalized lymphopenia by improved definition of the irradiated volume.^169^ Although the underlying mechanisms remain to be elucidated, hypofractionated ablative RT (8–12.5 Gy/fraction, for two to three fractions) seems to be superior to single-dose RT in inducing an antitumor T-cell response and creating a favorable TME for maximal efficacy of checkpoint blockade in preclinical models (Table 6).^87,138,170,171^ A recent clinical trial reported the outcomes of 22 metastatic melanoma patients treated with ipilimumab and different RT regimens.^5^ Three patients experienced a sustained complete response and were treated with 50 Gy in 4 fractions, 24 Gy in 3 fractions or 40 Gy in 10 fractions, respectively. Finally, a retrospective analysis of 44 melanoma patients treated with RT and ipilimumab showed a significantly increased survival of patients treated with ablative as compared with patients treated with conventionally fractionated RT.^132^ However, conventionally or less hypofractionated RT may also synergize with immune-checkpoint therapy. A clinical trial combining GM-CSF and hypofractionated RT of 35 Gy in 10 fractions in 41 patients of several tumor types reported two CRs and six PRs.^8^ Moreover, conventionally fractionated RT synergized with anti-PD-L1 in different mouse models and induced the formation of antitumor immunological memory.^64,65^ Indeed, the effects of conventionally fractionated RT on IT efficacy may be underestimated due to the technical difficulties in applying many sequential RT doses to mice. Future studies should address this question, since conventionally fractionated RT remains the standard radiation regimen in many tumor types and stages. Hence, the limited number of reports calls for further investigation of the effects of different fractionation regimens on combined RT and IT.

**Table 6.** Comparison of different radiation regimens in combination with immune checkpoint therapy.

Study type	Immune checkpoint	Tumor type	Timepoint of IT before/after RT	Fractionation radiation dose	Conclusions	References
Retrospective clinical	CTLA-4	Melanoma	Before/after	1–5*5–22 Gy 5–20*2.3–4 Gy	Median OS 19.6 *versus* 10.2 months in patients with ablative *versus* patients with conventionally fractionated RT, respectively	Qin *et al*.^132^
Preclinical	CTLA-4	Breast (4T1)	+1, 4, 7 d	1*12 Gy 2*12 Gy	Fractionated RT is superior to single-dose RT.	Demaria *et al*.^81^
Preclinical	CTLA-4	Breast (TSA)	+0, 3, 6 d	1*8 Gy 1*30 Gy 3*8 Gy	Fractionated radiotherapy, but not single-dose radiotherapy, induces an abscopal effect.	Vanpouille-Box *et al*.^136^
Preclinical	CTLA-4	Breast (TSA) MCA38 (colorectal)	+2, 5, 8 d	1*20 Gy 3*8 Gy 5*6 Gy	Fractionated radiotherapy is superior to single-dose radiotherapy when combined with anti-CTLA-4 in two mouse models; a more hypofractionated regimen of 3*8 Gy is superior to a less hypofractionated regimen of 5*6 Gy when combined with CTLA-4	Dewan *et al*.^138^
Preclinical	PD-L1	Pancreatic (Pan02)	+0 d	1*12 Gy 5*3 Gy	Fractionated and single dose equally synergize with anti-PD-L1.	Azad *et al*.^64^
Preclinical	PD-1 CD137	Breast (AT-3)	+0 d	1*12 Gy 4*5 Gy 4*4 Gy	Both single dose and fractionated RT + IT synergize with anti-PD-1 and anti-CD137.	Verbrugge *et al*.^31^

Representative clinical and preclinical *in vivo* studies comparing different radiation regimens in combination with immune checkpoint therapy. Characteristics of the studies with the main conclusions are included.

CTLA-4, cytotoxic T-lymphocyte associated protein 4; d, days; Gy, Gray; OS, median overall survival; IT, immunotherapy; PD-1, programmed cell death 1; PD-L1, CD274 molecule; RT, radiotherapy.

### Irradiation volume

Another important factor which could impact the outcome of combined RTs and ITs is the irradiated volume. Most preclinical and clinical studies combining RT and IT focused on local RT. Local RT can either be administered by external-beam RT or brachytherapy and both approaches can induce abscopal responses.^3,4,172^ However, A adoptive T or NK cell transfers (ACTs) not only benefit from local RT, but also from TBI and other lymphodepleting regimens.^173^ Preclinical studies revealed several effects of TBI on ACTs, including enhanced engraftment, increased proliferation and effector function of transferred lymphocytes.^85,107,109^ Besides, both TBI and local RT enhance T-cell infiltration or tumor susceptibility to T-cell-mediated lysis, resulting in higher antitumor efficacy of ACTs. It is therefore compelling to assume that combining TBI with a local booster dose could optimally enhance ACTs. Nevertheless, in cancer patients, chemotherapy is generally used instead of TBI to enhance ACT engraftment. A recent phase III clinical trial showed no benefit of adding TBI to an adoptive T-cell transfer after a preconditioning chemotherapy regimen, suggesting that the latter is sufficient for effective lympho-depletion.^174^ However, this could be different in hematopoietic cancers where cells frequently spread to the bone-marrow and where TBI constitutes a standard treatment before hematopoietic stem-cell transplantation.

In ITs relying on priming of tumor-reactive T cells such as checkpoint inhibitors, radiation or surgical removal of the tumor-draining lymph nodes could impede therapeutic efficacy. Sparing macroscopically nonaffected tumor-draining lymph nodes from RT may add benefit to patient survival and its combination with IT needs to be prospectively addressed in clinical trials.^18^ Moreover, the radiation field should not include large skin areas, since Treg cells can be primed by activation of Langerhans cells residing in the irradiated skin.^175^ Therefore, irradiating the tumor from few angles could be superior to conventional three-dimensional conformal RT.

### Timing

Timing is another critical factor when applying combined RT and IT. A retrospective analysis revealed that in patients undergoing combined RT and IT for brain metastasis, timing of RT strongly correlated with patient outcome. Interestingly, patients receiving concurrent RT and ipilimumab had a longer overall survival than patients receiving ipilimumab before or after RT.^124,126^ Moreover, a phase I clinical trial of concurrent RT and ipilimumab in 22 patients with metastatic melanoma reported three CRs, whereas no CRs were observed in a clinical trial of sequential RT and ipilimumab in metastatic melanoma.^5,69^ This notion was further substantiated by studies in syngeneic mouse models confirming the superiority of concurrent *versus* consecutive PD-L1 or CTLA-4 checkpoint inhibition.^140^ Likewise, most preclinical and clinical studies administered checkpoint inhibitors concurrently with RT, which appears to be the preferred timing schedule, as recently supported by mathematical modeling^176^ (Table 5).

As opposed to checkpoint inhibitors, ACTs were not delivered concurrently but sequentially, directly after RT, because adoptively transferred cells may be impaired or killed by concurrent irradiation. Importantly, the window for effective adoptive transfer after RT appears to be narrow. In a syngeneic mouse model, T cells rejected all tumors when they were adoptively transferred 2 days after RT but did not reject any tumors when they were transferred 4 days after RT.^48^ This might suggest that ACTs mainly benefit from early effects of RT, such as the induction of chemokines, cytokines and immunogenic modulation of DAMPs on the tumor cells (Tables 2, 4). Of note, animal models often progress considerably faster than cancer patients, rendering delayed spaced regimens unfeasible. These must therefore be evaluated differently.

### Additional factors influencing combined radio- and immunotherapy

Immunogenicity of the tumor is a critical factor that needs to be considered. The tumor type may heavily influence the response to combined RT and IT. Priming of tumor antigen-specific T cells in cancer patients after RT was frequently observed in colorectal cancer patients but less frequently in prostate cancer patients. In this sense, prostate cancer is believed to be a poorly immunogenic cancer entity.^177^ Moreover, the upregulation of immunogenic surface molecules after RT is confined to a fraction of cell lines.^57^ Nevertheless, there are few comprehensive studies to generalize these findings.

The patient’s immune status should be considered when planning RT and IT combination trials.^19,116^ It is conceivable that immune parameters could also be employed to predict the response of patients to combined RT and IT but this remains to be evaluated. In this line, patients responding to combined RT and IT showed a lower number of tumor-infiltrating MDSCs and a higher frequency of T cells with an activated effector memory phenotype.^178,179^ Moreover, a recent randomized controlled clinical trial in castration-resistant prostate cancer patients indicated that patients with features of less advanced disease benefited more from RT plus ipilimumab compared with RT alone than patients with advanced disease,^123^ which could be explained by a less advanced TME with lower suppression of antitumor immunity.

Concurrent treatments and medication of the patient could alter the radiation-induced immune response and should therefore be considered. Surgery greatly diminished antigen abundance and impeded antitumor immunity in a preclinical mouse model of fibrosarcoma.^149^ Corticoids and antibiotics are frequently administered after RT to treat complications such as radiation-induced emesis, pneumonitis and infections. Dexamethasone entails immunosuppressant effects and ciprofloxacin abrogates the radiation-induced translocation of gut microbiota resulting in limited efficacy of RT or combined RT and IT in mice.^27,110^ Despite the fact that some cytotoxic drugs alone can induce antitumor immune responses,^119^ they can either have beneficial or detrimental effects when added to combined RT and IT. LD chemotherapy administered before initiation of combined RT and IT can be beneficial by lowering systemic Treg or MDSC numbers.^158^ By contrast, full-dose chemotherapy administered after initiation of combined RT and IT inhibits the proliferation of tumor-reactive T cells. Thus, the type of concurrent medication and its effects on the immune system should be considered when combining RT and IT.

## Conclusion

Preclinical studies have been of much importance elucidating new mechanisms of RT on the immune system. But more translational studies are needed to evaluate whether RT can enhance the priming of tumor-reactive T cells in large cohorts of patients and whether they induce CD8 and CD4 immunological memory. Even though combined treatments have shown considerable promise, many patients do not respond to combined RT and IT, which means that further mechanistic preclinical studies are needed to unveil novel clinical approaches combining these two treatments.^106,121,123^

Radiation dose, fractionation and timing must all be optimized to enhance IT in each tumor type and stage, and these should be established in future clinical trials. The complexity of this question would require systematic approaches in experimental models and in patients. In our opinion, consensus on novel radiological response criteria are needed to capture benefit in terms of local *versus* abscopal/systemic responses to radioimmunotherapy. Ultimate evidence in randomized clinical trials is unlikely to be available in the next 5 years.

The wide implementation of modern RT techniques such as intensity-modulated radiation therapy and four-dimensional conformal radiation therapy facilitates the clinical translation of combined RT and IT. The high radiation doses frequently needed for enhancing IT can be administered with high precision. Moreover, detailed analyses of the effects of emerging RT techniques such as proton and heavy ion therapy on the immune system remain to be addressed. Inflammatory responses post-RT can cause serious side effects such as pneumonitis, myocarditis and fibrosis. It is currently unknown how enhanced immune reactivity after RT and IT may impact these adverse events and how they can be prevented without limiting the antitumor immune response. Finally, a scenario, which has so far been largely ignored in preclinical studies, is the combination of RT, IT and surgery either in the adjuvant or neoadjuvant setting. Future preclinical research should account for this combination of great clinical importance and identify its distinct immunological features such as a highly diminished tumor antigen load in the adjuvant setting.

Brachytherapy offers opportunity for local delivery of IT agents in addition to the local instigation of RT. Indeed, the combination of intraoperative RT and IT also offers opportunities that remain unexplored at this point.

The insights obtained from studying the effects of RT on the immune system could also lead to the development of new ITs acting synergistically with RT. Given the complexity of immunological changes in the TME postirradiation, approaches using computational tools and systems biology will gain more importance in the field and shed light on complex spatio-temporal players of the TME post-RT. This can ultimately lead to the development of novel and more complex combination therapies,^180^ which could overcome resistance to RT plus single/dual-agent immunotherapies and which may therefore be applicable in complex settings such as at multimetastatic stages. Abscopal effects after RT represent one of the most exciting themes, and a better understanding of their mechanistic basis in multiple tumors and stages could lead to a paradigm shift in radiation oncology that could turn a local mode of cancer treatment into a systemic one.
